# Babesiosis: An Atypical Cause of Respiratory Failure

**DOI:** 10.7759/cureus.39028

**Published:** 2023-05-15

**Authors:** Jared R Muench, Pinky Jha, Antoni Wojtkowski

**Affiliations:** 1 Internal Medicine, Medical College of Wisconsin, Milwaukee, USA

**Keywords:** acute respiratory distress syndrome (ards), non-cardiogenic pulmonary edema, ixodes tick, asplenia, tick-borne disease, parasitemia, hemolytic anemia, pulmonary manifestations, acute respiratory failure, babesiosis

## Abstract

Babesiosis is a parasitic infection of the *Babesia* protozoa, which has been increasing in incidence in endemic areas of the United States. Symptoms of babesiosis can occur on a wide spectrum, from a mild flu-like illness to a fulminant disease course. Known complications of severe cases include intravascular hemolytic anemia and may involve the coagulation system, heart, spleen, kidneys, and in some cases, the lungs. This case report describes an 81-year-old, asplenic female in northern Wisconsin who presented to a hospital with shortness of breath and a non-productive cough. Definitive diagnosis of babesiosis, which was made through both a nucleic acid panel and blood smear, was initially delayed given the rare pulmonary manifestation of babesiosis. When the lungs are involved in the disease course, non-cardiogenic pulmonary edema leading to acute respiratory distress syndrome is among the most commonly seen complications. The pathophysiology of pulmonary involvement has not been made entirely clear but is most likely multifactorial, including the sequelae of changes to both the patient's red blood cells and pulmonary vasculature. This report highlights that atypical tick-borne illnesses like babesiosis should be considered as a cause of acute respiratory failure, particularly in the setting of sepsis and fever. The threshold for parasitic testing should be low in patients in endemic regions with risk factors, including increased age and history of asplenia, as babesiosis frequently has no localizing symptoms to suggest a protozoan infection. As babesiosis incidence continues to rise, prompt diagnosis and proper treatment can prevent severe complications and death in patients.

## Introduction

Babesiosis is a primarily tick-borne illness caused by intraerythrocytic protozoa of the *Babesia *genus (most commonly *Babesia microti* transmitted by the *Ixodes scapularis* tick). Endemic regions in the United States with tick-borne transmission include the Northeast (e.g., New England, New York, New Jersey) and the upper Midwest (e.g., Wisconsin, Minnesota). Annual national babesiosis incidence has risen to 8.6 cases per million persons, and the total number of cases per year has more than doubled since 2012, totaling over 2400 cases in 2019 [1]. As human habitats continue to expand into wooded areas with high tick density and as community recognition and awareness of the disease increases, babesiosis is likely to remain a disease of growing concern.

Symptoms of babesiosis can occur on a spectrum from asymptomatic to a fulminant illness, progressing even to coma and death. Typical signs and symptoms include fatigue, fever, nausea, myalgias, and headache, but may involve complications of the coagulation system, heart, spleen, and kidneys. The lungs are infrequently affected in babesiosis, but cases may be severe when their involvement is seen. Here, a rare case of babesiosis complicated by pulmonary manifestations in an 81-year-old asplenic female is described. This case report was previously presented as a poster at the 2022 American College of Physicians Meeting on April 30, 2022.

## Case presentation

**Figure 1:**
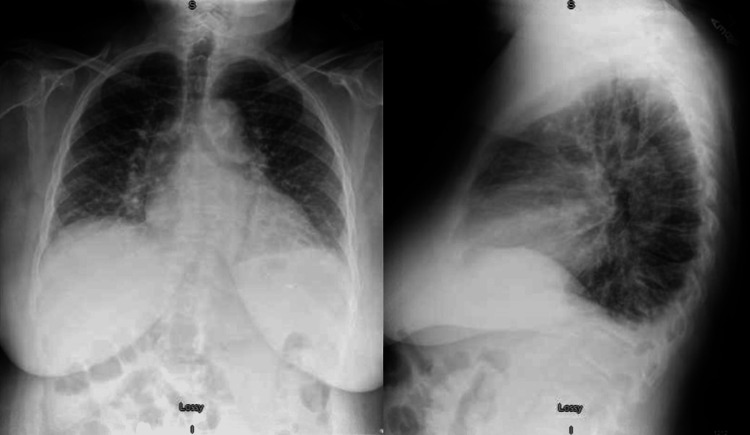
Chest X-ray on admission demonstrated pulmonary edema and borderline cardiomegaly, but without pleural effusion

An 81-year-old female living in northern Wisconsin with a history of diastolic heart failure and asplenia (s/p splenectomy in 1999 for splenic artery aneurysm) presented to an outside hospital for a week-long course of shortness of breath with a nonproductive cough. She also noted frontal headaches, chills, and poor appetite for the past 10 days. On admission, the patient was febrile to 102.9F with a respiratory rate of 24 and found to be in acute hypoxic respiratory failure requiring three to four liters of supplemental oxygen. Physical exam was unremarkable apart from diminished breath sounds bilaterally. Labs on presentation were overall reassuring without concern for organ damage. A comprehensive metabolic panel (CMP) showed electrolytes within normal limits (apart from low sodium of 128 mEq/L), normal blood urea nitrogen (BUN) of 17 mg/dL, and creatinine (Cr) of 0.88 mg/dL at baseline. Liver function tests were mildly elevated with aspartate transaminase (AST) and alanine transaminase (ALT) of 59 and 41 IU/L, respectively, although total bilirubin was not elevated at 1.3 mg/dL. The complete blood count (CBC) showed mild anemia (hemoglobin and hematocrit (H&H) of 11.6 g/dL and 33.5%) with normal mean corpuscular volume (MCV) of 92.5 fL, no leukocytosis (WBC 9.3 k/microliter), and platelet levels at the low end of normal (PLT 145 k/microliter). Coagulation studies were normal, with a prothrombin time (PT) of 10.7 seconds and a partial thromboplastin time (PTT) of 24.0 seconds. Lactate was not elevated at 1.0 mmol/L, although procalcitonin was elevated to 0.71 ng/mL. Urinalysis and cultures were negative. NT-pro BNP was elevated from baseline to 2,583 pg/mL. Initial chest X-ray demonstrated pulmonary edema and borderline cardiomegaly without pleural effusions (Figure 1). A CT pulmonary embolism protocol was also performed but was negative. Given the initial concern for community-acquired pneumonia versus heart failure exacerbation, the patient was started on ceftriaxone and azithromycin, as well as furosemide.

**Figure 2:**
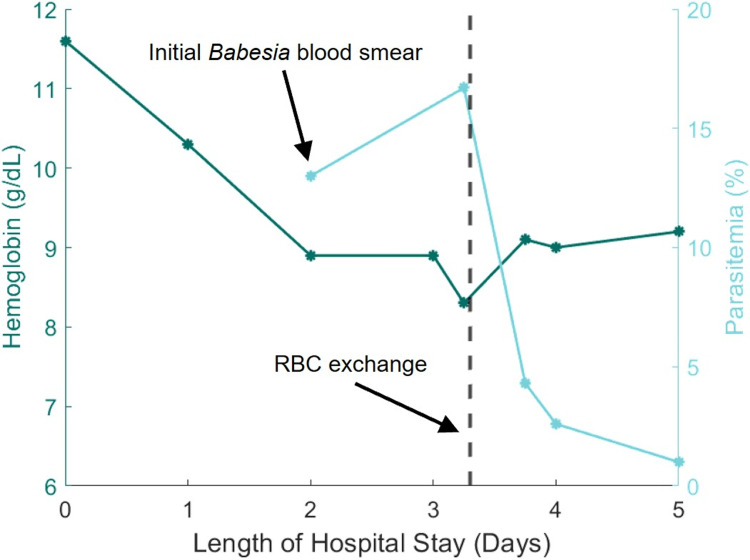
Trends in patient hemoglobin (g/dL) and parasitemia (%) throughout the patient's hospital course

Despite diuresis, antibiotic therapy, and oxygen support, the patient continued to clinically deteriorate over the next 48 hours. She experienced multiple episodes of hypotension and a nearly three-gram per deciliter drop in hemoglobin from admission (11.6 to 8.9 g/dL), without evidence of active bleeding (Figure 2). Therefore, concern for other atypical infections was raised. Given the patient's hemolytic anemia (i.e., hemoglobin 8.9 g/dL, lactate dehydrogenase 694 IU/L, haptoglobin <10 mg/dL, reticulocyte count 5.8%) in the setting of her residence in northern Wisconsin, tick-borne illness was of primary concern. Tick-borne pathogen nucleic acid panel was ordered and returned positive for *Babesia microti*. A manual review of the blood smear was also performed, and intracellular organisms were visualized with severe parasitemia of 13%.

Given this patient had severe symptoms and experienced complications, she was subsequently treated with standard therapy of PO atovaquone 750mg BID and IV azithromycin 500mg q24hr for a total course of 10 days [2]. Doxycycline was also added to cover for possible co-infection with other diseases transmitted by the *Ixodes *tick (e.g., Lyme disease, ehrlichiosis, anaplasmosis). Given the severity of the illness and the high degree of parasitemia, the patient was transferred between hospitals for a higher level of care and red blood cell exchange transfusion [2]. Upon arrival, repeat labs showed worsening parasitemia to 17%, and hematology was consulted for initiation and management of red blood cell exchange. Following the exchange transfusion on hospital day four, the parasite burden dramatically decreased to less than one percent, and hemoglobin began to rebound (Figure 2). A blood smear was performed each morning to monitor the parasite burden and efficacy of treatment. Because her parasitemia remained low, her anemia continued to improve, and she showed no further signs of organ dysfunction, repeat exchange transfusion was not warranted. She was gradually weaned back to room air after the transfusion and maintained saturations at 94-96% without signs of ongoing respiratory distress. The patient was discharged on hospital day five with oral atovaquone 750mg BID and azithromycin 500mg QD to complete the full 10-day course. A CBC was completed in the outpatient setting to monitor her hemoglobin, which had returned to 11.0 g/dL. She was seen in follow-up by Infectious Disease one week after discharge from the hospital and was noted to be doing well without further complications.

## Discussion

Presenting signs of dyspnea and nonproductive cough often elicit a differential diagnosis of pneumonia versus heart failure exacerbation vs. obstructive lung disease and are subsequently treated with a combination of therapies. However, in endemic regions, atypical tick-borne illnesses like babesiosis should be considered as a cause of acute respiratory failure, particularly in the setting of sepsis and fever. Although pulmonary manifestations of babesiosis are rare, non-cardiogenic pulmonary edema progressing to acute respiratory distress syndrome (ARDS) is among the most commonly seen pulmonary complications of the disease [3]. The exact pathogenesis has not been made entirely clear. However, *Babesia *infection is known to induce a pro-inflammatory host response and release of cytokines, producing a typical viral-like illness. In addition, these cytokines (e.g., TNF-α, IL-6) affect the pulmonary vasculature [4], likely contributing to fluid extravasation in the lungs. As with several of the manifestations of babesiosis, pulmonary symptoms may also be related to the intraerythrocytic nature of the pathogen. The *Babesia *parasite replicates within erythrocytes, damaging the membrane upon its exit and causing widespread intravascular hemolysis that may progress to hypoxemia. Furthermore, changes to the cell membrane cause decreased erythrocyte conformability and increased cytoadherence to vessel walls, leading to obstruction of pulmonary vascular flow and, ultimately, non-cardiogenic pulmonary edema and ARDS [5]. This mechanism of parasite sequestration and lung injury is known to be used by another intraerythrocytic organism - *Plasmodium falciparum*­ - which causes malaria [6].

*Babesia *infections can progress along a range of disease courses, from asymptomatic to fatal. Most patients present with signs and symptoms of a generalized illness, including fatigue, fever and chills, nausea or vomiting, myalgias or arthralgias, and headache. However, shortness of breath and/or cough has been reported as a presenting symptom in 15 to 33% of cases [7-9]. Many of these cases exhibit mild symptoms without major organ involvement and are suitable for outpatient treatment. However, hospitalization rates following a diagnosis of babesiosis, or while a definitive diagnosis is being made, may be as high as 63% [8]. While the median length of stay may be as low as five days [8], up to 25% of hospitalized patients may require care for greater than two weeks, with some patients being hospitalized for even longer than one month [7,9].

Presumably, longer lengths of stay may be due to the presence of complications throughout the disease course. Complications may develop in up to one of every two to three patients hospitalized for babesiosis [4,7,9]. Described complications include severe anemia, congestive heart failure, renal failure, and disseminated intravascular coagulation. However, ARDS is frequently among the most common complications in numerous studies, with reported prevalence as high as 20% [7-9], and even higher rates in ICU patients [10]. ARDS is a severe form of non-cardiogenic pulmonary edema caused by inflammatory-mediated increased capillary permeability in the lungs and has associated mortality rates exceeding 40% in severe cases [11]. Although this patient did not progress to ARDS and intubation was not indicated in her case, ventilatory support may be required in cases of severe babesiosis with pulmonary manifestations. The reported prevalence of intubation in these hospitalized patients ranges from 3 to 6% [8,9]. ICU level of care is not uncommon for these patients. A quarter of patients (i.e., 25.2%) required ICU care in a review of 139 cases of babesiosis in New York [9], while a similar review of 128 cases from northern Wisconsin saw a slightly lower percentage (i.e., 11.7%) of patients needing intensive care [8]. Ultimately, reported fatality rates range from 6-9% among hospitalized patients [4,9], while rates for babesiosis patients in the ICU, specifically with ARDS, may be as high as nearly 40% [10].

This patient demonstrated a high degree of parasitemia - as high as 17% on the third day of hospitalization - and also demonstrated severe clinical symptoms. A previous study in Long Island, New York, found that complications as a whole (including congestive heart failure, kidney failure, disseminated intravascular coagulation, and acute respiratory failure) were strongly associated with severe anemia (hemoglobin <10 g/dL) and, to a lesser degree, with high-grade parasitemia (≥10 percent) [7]. However, the association between the degree of parasitemia and clinical severity has actually been shown to be weak. In a study exploring the efficacy of red blood cell exchange for treating babesiosis, four patients expired, three of whom had parasitemia levels ≤0.3%. This study also found that parasitemia level following red blood cell exchange was not correlated with hospital length of stay [12]. Despite the small group size, this indicates that a high parasite burden is not a requisite for a severe clinical course. In the previously-mentioned Long Island study, there were no risk factors analyzed that were specifically associated with an increased risk of acute respiratory failure. In fact, numerous cases of ARDS following babesiosis have been reported with parasitemia levels <1% and have also been noted to develop only after the initiation of antibiotics [13]. Current hypotheses propose that by initiating anti-*Babesia *treatment, the destruction of *Babesia *organisms activates an intense inflammatory response, thereby releasing mediators leading to lung injury and the development of ARDS despite low parasite burden [3,14].

Several known risk factors predispose patients to severe cases of babesiosis. As in many infections, immune status plays a key role in determining the severity of disease, as immunocompromised patients are more likely to experience more severe symptoms of babesiosis than immunocompetent patients [4]. However, other risk factors for severe disease are known to exist, two of which likely contributed to the disease in this patient. The absence of a spleen is a major risk factor for severe symptoms. In asplenic individuals, as in this patient, both *Babesia*-infected erythrocytes and erythrocyte fragments are not cleared from circulation [15], leading to a more fulminant disease course and many of the organ system complications described above. Finally, elderly individuals (i.e., >50 years of age) often experience greater severity of symptoms as a likely result of the age-related decline in cellular immunity [4]. Even in the setting of multiple risk factors, one's index of suspicion for babesiosis must be high, as the disease frequently has no localizing symptoms to suggest a protozoan infection.

## Conclusions

Babesiosis and other atypical parasitic infections should be considered as a differential diagnosis in all patients presenting with respiratory failure in the setting of sepsis and fever in endemic regions. One's index of suspicion must be high, as babesiosis frequently has no localizing symptoms to suggest a protozoan infection. The threshold for parasitic testing should be even lower in patients with risk factors, including increased age and history of asplenia. As babesiosis incidence continues to rise, prompt diagnosis and proper treatment can minimize severe complications and death in patients.
